# The White Blood Cell Count to Mean Platelet Volume Ratio for the Prediction of Chronic Limb-Threatening Ischemia in Lower Extremity Artery Disease

**DOI:** 10.3390/jcm8101593

**Published:** 2019-10-02

**Authors:** Katharina Guetl, Reinhard Bernd Raggam, Viktoria Muster, Paul Gressenberger, Jovan Vujic, Alexander Avian, Franz Hafner, Martin Wehrschuetz, Marianne Brodmann, Thomas Gary

**Affiliations:** 1 Division of Vascular Medicine, Department of Internal Medicine, Medical University of Graz, 8036 Graz, Austria; reinhard.raggam@medunigraz.at (R.B.R.); viktoria.muster@medunigraz.at (V.M.); paul.gressenberger@medunigraz.at (P.G.); jovanvuj@gmail.com (J.V.); franz.hafner@medunigraz.at (F.H.); marianne.brodmann@medunigraz.at (M.B.); thomas.gary@medunigraz.at (T.G.); 2 Institute for Medical Informatics, Statistics and Documentation, Medical University of Graz, 8036 Graz, Austria; alexander.avian@medunigraz.at; 3 Department of Radiology, Medical University of Graz, 8036 Graz, Austria; martin.wehrschuetz@gmail.com

**Keywords:** atherosclerosis, peripheral arterial disease, lower extremity artery disease, chronic limb-threatening ischemia, platelets, white blood cells

## Abstract

Background: The white blood cell count to mean platelet volume ratio (WMR) is increasingly gaining importance as a promising prognostic marker in atherosclerotic disease, but data on lower extremity artery disease (LEAD) are not yet available. The principle aim of this study was to assess the association of the WMR with the occurrence of CLTI (chronic limb-threatening ischemia) as the most advanced stage of disease. Methods: This study was performed as a retrospective analysis on 2121 patients with a diagnosis of LEAD. Patients were admitted to the hospital for the reason of LEAD and received conservative or endovascular treatment. Blood sampling, in order to obtain the required values for this analysis, was implemented at admission. Statistical analysis was conducted by univariate regression in a first step and, in case of significance, by multivariate regression additionally. Results: Multivariate regression revealed an increased WMR (*p* < 0.001, OR (95%CI) 2.258 (1.460–3.492)), but also advanced age (*p* < 0.001, OR (95%CI) 1.050 (1.040–1.061)), increased CRP (*p* < 0.001, OR (95%CI) 1.010 (1.007–1.014)), and diabetes (*p* < 0.001, OR (95%CI) 2.386 (1.933–2.946)) as independent predictors for CLTI. Conclusions: The WMR presents an easily obtainable and cost-effective parameter to identify LEAD patients at high risk for CLTI.

## 1. Introduction

Peripheral artery disease (PAD) is common and encompasses numerous entities of vascular disorders in the arterial part of the vascular system, except for intracranial and coronary arteries. Atherosclerosis is the main cause of PAD [1]. Worldwide, over 200 million people are living with a diagnosis of PAD [2]. Major risk factors for the development of PAD comprise age, smoking, hypertension, diabetes, dyslipidemia, and a history of cardiovascular disease [3]. Lower extremity artery disease (LEAD) affects arteries in the lower limbs and is associated with increasing vascular morbidity and mortality [4]. Chronic limb-threatening ischemia (CLTI) is the most advanced form of LEAD. The diagnosis of CLTI is made clinically and requires the presence of ischemic-induced rest pain, ulcers, or gangrene [5]. Although treatment strategies are improving, amputation rates in patients with CLTI still remain high [6]. Thus, early diagnosis and initiation of therapy are required to avoid disease progression and to improve outcome [7].

Nowadays, white blood cells and platelets are acknowledged to play a crucial role in atherothrombosis and atherosclerosis. Atherosclerosis is an inflammatory disease that is characterized by numerous inflammatory processes in the arterial wall [8]. In PAD patients, the total white blood cell (WBC) count and markers of inflammation in general are increased, as compared to patients without PAD [9,10]. Besides that, platelets cover a central position in the process of atherothrombosis and in the progression of atherosclerotic plaque formation [11], but they are also involved in the initiation of atherosclerosis by binding to inflamed but otherwise intact endothelium [12,13].

Based upon this knowledge about platelets and leukocytes in atherosclerotic and atherothrombotic disease, recent studies investigated and confirmed an association between the platelet-to-lymphocyte ratio (PLR), the neutrophil-to-lymphocyte ratio (NLR), and the lymphocyte-to-monocyte ratio (LMR) with CLTI and other cardiovascular endpoints in patients with LEAD [14,15,16]. Moreover, an analysis by Rief et al. [17] lately pointed out a relation between decreasing mean platelet volume (MPV) and CLTI. Apart from that, several previous studies have already targeted platelet parameters and white blood cell count parameters, as well as combinations of them, in order to investigate their role as prognostic markers in patients with coronary artery disease (CAD) [18,19,20,21,22] and cerebrovascular disease [23,24,25,26].

Focusing on the white blood cell count to mean platelet volume ratio (WMR) as a promising prognostic marker for cardiovascular outcome in patients with atherosclerotic disease, there is little data available on patients with CAD [27,28], but, to date, the role of the WMR in LEAD and also in cerebrovascular disease remains unexamined.

The principle aim of this current analysis was to ostensibly determine the association of the WMR with CLTI in LEAD. A rise in the WMR may result either from an increase in the WBC count or from a decrease in the MPV. We hypothesized that a higher WMR will be able to indicate patients at high risk for the occurrence of CLTI, but also for further cardiovascular outcome events, such as myocardial infarction (MCI) and stroke. We believe that identification of a high-risk population is of great importance, as these patients may benefit from intensified treatment options and aggressive management of cardiovascular risk factors, in order to prevent cardiovascular mortality and morbidity.

## 2. Materials and Methods

### 2.1. Study Design

Our study was performed as a retrospective analysis that included a total of 2121 patients. Patients met the inclusion criteria when they were admitted to the hospital for the reason of LEAD at the Division of Angiology, Medical University of Graz, in a time period between 2005 and 2010. The diagnosis of LEAD was assigned at our outpatient clinic by means of clinical examination, ankle brachial index [29], and color-encoded duplex sonography, according to the revised criteria as described in the Trans-Atlantic Inter-Society Consensus Document on Management of Peripheral Arterial Disease (TASC II) [7]. The severity of LEAD was categorized by the Fontaine classification [30]. CLTI, as referring to classes 3 and 4, following the Fontaine classification, was defined by the presence of ischemic-induced rest pain and/or skin defects, which comprise ulcers and gangrene [5]. Patients received either conservative treatment or endovascular therapy. Major conservative treatment options were improvement of cardiovascular risk factors, optimized medical treatment, and exercise therapy. Selected patients received prostanoid infusion therapy. Patients’ data for this analysis—including medical records with a focus on comorbidities, previous cardiovascular events, and the presence of cardiovascular risk factors—were obtained when patients were admitted to the hospital. Cardiovascular outcome events of interest for this analysis encompass CLTI, MCI, and stroke. Potential prognostic markers evaluated in this study for their association with the occurrence of cardiovascular outcome events were age, C-reactive protein (CRP), sex, diabetes, antiplatelet treatment with clopidogrel and/or aspirin, the WMR, and thrombocytosis. Blood samples for laboratory parameter measurements were taken at a single time in the fasting condition at the time of admission to the ward. No exclusion criteria were defined, as we did not expect any conditions and/or comorbidities to have a major influence on the outcome of this analysis.

### 2.2. Ethical Approval

Ethical approval for this study was administered by the International Review Board, Medical University of Graz, Austria (IRB Number 24-506 ex 11/12). Neither written informed consent nor verbal consent were required, as this analysis was performed retrospectively.

### 2.3. Statistical Analysis

Data are given as mean and standard deviation (SD) or median and interquartile range (IQR) for continuous data, and as a frequency for categorical data. Potential prognostic parameters—as mentioned above in the section of this manuscript describing the study design—were analyzed by univariate logistic regression to evaluate their association with endpoints of CLTI, MCI, and stroke. Variables showing significance in the univariate regression analysis were selected for the multivariate logistic regression. Variables in the final model were selected with a backward stepwise procedure. The decision to remove variables was based on a likelihood-ratio test. Results from the regression analysis are expressed as p-value and odds ratio (OR) with a 95% confidence interval (CI). A *p*-value of less than 5% was considered significant. Data were analyzed with SPSS 23.0.0.2 (SPSS Inc., Chicago, IL, USA).

## 3. Results

### 3.1. Patient Characteristics

A total of 2121 patients with a diagnosis of LEAD were included in this current analysis. Patients were predominately male (59.6% versus 40.4%), and the mean age was 69.3 ± 12.0. Regarding cardiovascular risk factors, arterial hypertension was found in 81.5% of patients, one-third of patients had a diagnosis of diabetes (33.6%), and 53.8% had a history of smoking. Concomitant CAD and cerebrovascular disease were present in 35.1% and 68.8% of patients, respectively. After assessing cardiovascular outcome events in this study cohort, it was found that 4.3% demonstrated prior MCI, 7.9% experienced prior stroke, and 32% presented CLTI. All patient characteristics are outlined in Table 1.

### 3.2. Chronic Limb-Threatening Ischemia

The univariate regression analysis showed a significant association of advanced age (*p* < 0.001, OR (95%CI) 1.052 (1.042–1.061)), increased CRP (*p* < 0.001, OR (95%CI) 1.015 (1.011–1.018)), male sex (*p* < 0.001, OR (95%CI) 1.4 (1.164–1.684)), and the presence of diabetes (*p* < 0.001, OR (95%CI) 2.723 (2.250–3.296)) with the occurrence of CLTI. Furthermore, a rise in risk for CLTI was found in patients without antiplatelet treatment with either clopidogrel (*p* = 0.004, OR (95%CI) 0.764 (0.634–0.919)) or aspirin (*p* = 0.003, OR (95%CI) 0.758 (0.630–0.913)), but also in patients with heightened WMR (*p* < 0.001, OR (95%CI) 2.915 (2.029–4.189)) and thrombocytosis (*p* < 0.001, OR (95%CI) 2.765 (1.776–4.306)). The multivariate logistic regression analysis subsequently revealed advanced age (*p* < 0.001, OR (95%CI) 1.050 (1.040–1.061)), increased CRP (*p* < 0.001, OR (95%CI) 1.010 (1.007–1.014)), the presence of diabetes (*p* < 0.001, OR (95%CI) 2.386 (1.933–2.946)), and heightened WMR (*p* < 0.001, OR (95%CI) 2.258 (1.460–3.492)) as significant predictors for CLTI (Table 2).

### 3.3. Myocardial Infarction

The univariate regression analysis spawned a significant association for prior MCI with age (*p* = 0.007, OR (95%CI) 1.026 (1.007–1.046)), CRP (*p* = 0.001, OR (95%CI) 1.006 (1.002–1.010)), diabetes (*p* = 0.004, OR (95%CI) 1.868 (1.228–2.841)), and with the absence of clopidogrel treatment (*p* = 0.016, OR (95%CI) 1.675 (1.099–2.553)). All of those components found as significant predictors in univariate regression pointed out significant results in the multivariate regression analysis, as well (age, *p* = 0.008, OR (95%CI) 1.028 (1.007–1.048); CRP, *p* = 0.005 OR (95%CI) 1.747 (1.135–2.689); diabetes, *p* = 0.011, OR (95%CI) 1.747 (1.135–2.689); and absence of clopidogrel treatment, *p* = 0.002, OR (95%CI) 1.962 (1.268–3.036)) (Table 3).

### 3.4. Stroke

In the univariate regression analysis, the endpoint of stroke was significantly associated with increased CRP (*p* = 0.002, OR (95%CI) 1.005 (1.002–1.008)), absence of aspirin (*p* = 0.001, OR (95%CI) 0.585 (0.427–0.803)), absence of clopidogrel (*p* = 0.028, OR (95%CI) 1.425 (1.039–1.954)), and heightened WMR (*p* = 031, OR (95%CI) 1.836 (1.056–3.192)). The multivariate regression approved the absence of aspirin (*p* = 0.009, OR (95%CI) 0.643 (0.461–0.896)), the absence of clopidogrel (*p* = 0.029, OR (95%CI) 1.449 (1.039–2.021)), and an increased WMR (*p* = 0.019, OR (95%CI) 1.943 (1.113–3.392)) as independent predictors for stroke, but CRP levels failed to have significance (Table 4). Interestingly, no significant association was found for the endpoints of myocardial infarction and stroke with thrombocytosis.

## 4. Discussion

In this retrospective study, we were able to demonstrate a statistically significant association of an elevated WMR with the occurrence of CLTI in LEAD patients. The relation between increased WMR levels and prior stroke reached significance, as well.

In recent years, numerous predictors for identifying LEAD patients being at high risk for CLTI were published. An increase in the PLR and an increase in the NLR are established markers for predicting the risk of CLTI and other vascular endpoints [14,15]. Tagoslu et al. showed that both the PLR and the NLR offer relevant information regarding limb survival and risk of amputation in CLTI [31,32]. Apart from that, a decrease in the MPV and in the LMR were shown to be significantly associated with the occurrence of CLTI [16,17]. All of these risk models are based on the knowledge about platelets and leukocytes playing a crucial role in the development and aggravation of atherosclerosis and atherothrombosis.

The influence of inflammatory processes and, thus, of white blood cells on atherosclerotic disease is widely recognized [33]. In 2014, Martin et al. published a review postulating a positive association of the WBC count with mortality rates and with the occurrence of major adverse events in PAD patients [34]. Regarding platelets in the field of atherosclerosis and atherothrombosis, their role covers not only participation in clot formation, but also involvement in inflammatory processes. In addition, platelets interact with endothelial cells and leukocytes, and they release inflammatory agents [35].

The WMR, as deriving from blood values of the WBC count and the MPV, presents a promising prognostic marker for cardiovascular outcome events. Since reflecting both cornerstone components —white blood cells and platelets—in the field of development and progression of atherosclerotic disease, we expect the WMR to present a favorable and superior prognostic marker.

Some data do exist about the WMR as a prognostic marker in CAD. In 2015, Dehghani et al. [28] investigated the WMR as a prognostic factor for cardiovascular outcome for the first time. In a prospective study design, the elevation in WMR levels at baseline was significantly associated with the incidence of major adverse cardiac events (MACEs) in a long-term follow-up in patients with non-ST segment elevation acute coronary syndrome [28]. Cicek et al. [27] recently focused on patients with ST segment elevation myocardial infarction and demonstrated an association of an elevated WMR with the occurrence of MACEs, and also with worse outcome in short- and long-term follow-up [27].

In our current investigation, we were able to point out a statistically significant association between the WMR and the occurrence of CLTI, but also between the WMR and stroke. There was no significance found for the endpoint of MCI in relation to elevated WMR levels. Hence, our findings are partly in contrast to the study results by Cicek et al. [27]. Even if LEAD and CAD are both mainly caused by atherosclerosis, patients with MCI differ from our patient cohort, and, therefore, both of these patient groups are not unrestrictedly comparable. In our analysis, the one and only criteria for inclusion was defined by the presence of LEAD, but CAD as a preexisting diagnosis was found only in one-third of our study population. Considering the fact that the study cohorts were not comparable might provide a possible explanation for deviating study results.

Our findings of elevated WMR levels in patients with CLTI and prior stroke may result from an increase in the WBC count and a decrease in the MPV, reflecting small platelets. Interestingly, previous studies about the MPV in CAD patients support an association of increasing MPV levels, reflecting larger platelets, with cardiovascular outcome events. For example, Goncalves et al. [36] suggested an elevated MPV as a strong independent predictor of outcome in CAD patients who underwent PCI, and Yang et al. [37] found elevated MPV levels in patients with confirmed restenosis in follow-up angiography after percutaneous transluminal coronary angioplasty (PTCA). The correlation of rising MPV levels with poor outcome in CAD patients may find an explanation in the fact that large-sized platelets are hemostatically more reactive than normal-sized platelets [37]. When regarding the role of the MPV as a predictor for CLTI in the patient cohort of our current analysis, low MPV was associated with high risk for CLTI [17]. This finding was recently supported when it was shown that small platelets respond with enhanced integrin activation after adenosine diphosphate (ADP) stimulation in comparison to large platelets, but large platelets perform aggregation significantly faster after collagen-induced stimulation than small platelets. Regarding the role of the thrombin receptor activating peptide (TRAP), high concentrations are associated with a more pronounced integrin-induced activation in small platelets, and low concentrations showed a higher level of activation in large platelets. This disproportional response of platelet activation is a new finding and not in line with previous studies, which always revealed stronger responses from large compared with small platelets [38].

The limitations of our study are, in particular, the retrospective design and the calculation of the WMR from a single blood sample. The retrospective design reflects a potential source of selection bias. In our region, different clinics take care of patients with LEAD. Since our clinic provides advanced treatment options as compared to others in the field of LEAD, we expect patients in more severe stages of disease to show up most frequently at our expert center. With regard to the point about how the WMR was calculated, it remains unclear whether a single blood sample mirrors an elevated WMR over time.

## 5. Conclusions

The WMR provides a rapid and easily obtainable, but also cost-effective, parameter to reliably identify LEAD patients at high risk for CLTI, together with other vascular endpoints, such as stroke. Selected patients may benefit from intensified medical treatment and the aggressive management of cardiovascular risk factors in order to prevent the progression of disease and a fatal outcome.

## Figures and Tables

**Table 1:** Patient characteristics.

Characteristics	Mean ± SD Median (IQR) *n* (%)
**Age (years)**	69.3 ± 12.0
**Male sex**	1266 (59.6%)
**Body mass index (BMI) (kg/m^2^)**	25 (23–28)
*Vascular risk factors*	
**Hypertension**	393 (18.5%)
**Diabetes**	713 (33.6%)
**Smoking history**	1142 (53.8%)
**Former smoking**	214 (10.1%)
*Vascular endpoints*	
**Myocardial infarction (MCI)**	92 (4.3%)
**Stroke**	168 (7.9%)
**Chronic limb-threatening ischemia (CLTI)**	680 (32%)
*Concomitant disease*	
**Heart failure**	185 (8.7)
**Coronary artery disease (CAD)**	746 (35.1%)
**Renal impairment**	606 (28.5%)
**Atrial fibrillation**	368 (17.3%)
**Cerebrovascular disease**	1458 (68.6%)

Data are given as mean ± standard deviation (SD), median (interquartile range; IQR), or frequency (*n*).

**Table 2:** Univariate and multivariate regression analysis for the endpoint of chronic limb-threatening ischemia (CLTI).

Endpoint CLTI	Mean ± SD Median (IQR) *n* (%)	Univariate Regression		Multivariate Regression	
		*p*-value	OR (95%CI)	*p*-value	OR (95%CI)
**Age (years)**	69.3 ± 12.0	**<0.001**	1.052 (1.042–1.061)	**<0.001**	1.050 (1.040–1.061)
**C-reactive protein (CRP) (mg/L)**	5.0 (2.0–13.6)	**<0.001**	1.015 (1.011–1.018)	**<0.001**	1.010 (1.007–1.014)
**Male sex**	1266 (59.6%)	**<0.001**	1.4 (1.164–1.684)		
**Diabetes**	713 (33.6%)	**<0.001**	2.723 (2.250–3.295)	**<0.001**	2.386 (1.933–2.946)
**Absence of clopidogrel**	1184 (55.7%)	**0.004**	0.764 (0.634–0.919)		
**Absence of aspirin**	830 (39.1%)	**0.003**	0.758 (0.630–0.913)		
**White blood cell count to** **mean platelet volume ratio (WMR)**	0.72 (0.59–0.88)	**<0.001**	2.915 (2.029–4.189)	**<0.001**	2.258 (1.460–3.492)
**Thrombocytosis**	83 (3.9%)	**<0.001**	2.765 (1.776–4.306)		

Data for the univariate and the multivariate regression are presented as odds ratio (OR) with a 95% confidence interval (CI), a p-value less of than 5% was considered significant.

**Table 3:** Univariate and multivariate regression analysis for the endpoint of myocardial infarction (MCI).

Endpoint MCI	Mean ± SD Median (IQR) *n* (%)	Univariate Regression		Multivariate Regression	
		*p*-value	OR (95%CI)	*p*-value	OR (95%CI)
**Age (years)**	69.3 ± 12.0	**0.007**	1.026 (1.007–1.046)	**0.008**	1.028 (1.007–1.048)
**CRP (mg/L)**	5.0 (2.0–13.6)	**0.001**	1.006 (1.002–1.010)	**0.005**	1.747 (1.135–2.689)
**Male sex**	1266 (59.6%)	0.366	0.818 (0.530–1.264)		
**Diabetes**	713 (33.6%)	**0.004**	1.868 (1.228–2.841)	**0.011**	1.747 (1.135–2.689)
**Absence of clopidogrel**	1184 (55.7%)	**0.016**	1.675 (1.099–2.553)	**0.002**	1.962 (1.268–3.036)
**Absence of aspirin**	830 (39.1%)	0.195	1.342 (0.860–2.094)		
**WMR**	0.72 (0.59–0.88)	0.415	1.387 (0.632–3.043)		
**Thrombocytosis**	83 (3.9%)	0.744	0.822 (0.255–2.655)		

**Table 4:** Univariate and multivariate regression analysis for the endpoint of stroke.

Endpoint Stroke	Mean ± SD Median (IQR) *n* (%)	Univariate Regression		Multivariate Regression	
		*p*-value	OR (95%CI)	*p*-value	OR (95%CI)
**Age (years)**	69.3 ± 12.0	0.162	1.010 (0.996–1.023)		
**CRP (mg/L)**	5.0 (2.0–13.6)	**0.002**	1.005 (1.002–1.008)		
**Male sex**	1266 (59.6%)	0.760	0.951 (0.689–1.313)		
**Diabetes**	713 (33.6%)	0.785	1.047 (0.752–1.459)		
**Absence of clopidogrel**	1184 (55.7%)	**0.028**	1.425 (1.039–1.954)	**0.029**	1.449 (1.039–2.021)
**Absence of aspirin**	830 (39.1%)	**0.001**	0.585 (0.427–0.803)	**0.009**	0.643 (0.461–0.896)
**WMR**	0.72 (0.59–0.88)	**0.031**	1.836 (1.056–3.192)	**0.019**	1.943 (1.113–3.392)
**Thrombocytosis**	83 (3.9%)	0.552	1.254 (0.594–2.646)		
